# OffScan: a universal and fast CRISPR off-target sites detection tool

**DOI:** 10.1186/s12864-019-6241-9

**Published:** 2020-03-05

**Authors:** Yingbo Cui, Xiangke Liao, Shaoliang Peng, Tao Tang, Chun Huang, Canqun Yang

**Affiliations:** 1 School of Computer, National University of Defense Technology, Changsha,, 410073 China; 2 National Supercomputing Center, Changsha, 410082 China; 3 College of Information Science and Engineering, Hunan University, Changsha, 410006 China

**Keywords:** CRISPR/Cas, sgRNA, Off-target, FM-index

## Abstract

**Background:**

The Type II clustered regularly interspaced short palindromic repeats (CRISPR) and CRISPR-associated proteins (Cas) is a powerful genome editing technology, which is more and more popular in gene function analysis. In CRISPR/Cas, RNA guides Cas nuclease to the target site to perform DNA modification.

**Results:**

The performance of CRISPR/Cas depends on well-designed single guide RNA (sgRNA). However, the off-target effect of sgRNA leads to undesired mutations in genome and limits the use of CRISPR/Cas. Here, we present OffScan, a universal and fast CRISPR off-target detection tool.

**Conclusions:**

OffScan is not limited by the number of mismatches and allows custom protospacer-adjacent motif (PAM), which is the target site by Cas protein. Besides, OffScan adopts the FM-index, which efficiently improves query speed and reduce memory consumption.

## Background

**Fig. 1:**
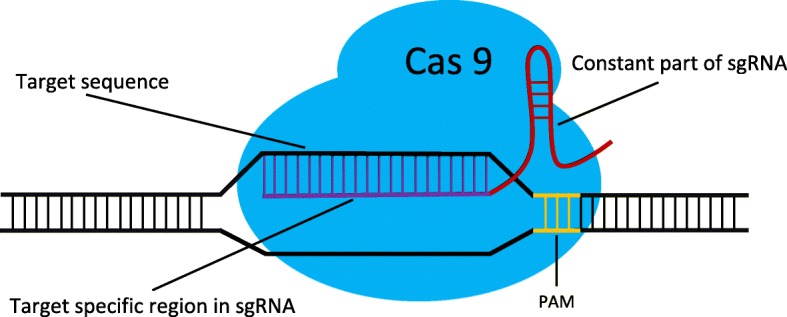
The CRISPR/Cas9 gene editing system

CRISPR/Cas is a powerful genome editing tool. When delivered into cells, as illustrated in Fig. 1, sgRNA will guide Cas nuclease to the desired DNA site and create a DNA double-strand break, the repair of which leads to a variety of DNA sequence modifications [1–3]. The performance of CRISPR/Cas is highly dependent on well-designed sgRNA. However, the off-target effect of sgRNA may lead to undesired mutations in the genome and limit the use of this technology. The off-target effect is caused by both sgRNA and Cas9. A few mismatches between the 5′ 20-nt sequence in sgRNA (the purple part in Fig. 1) and the target DNA sequence can be tolerated [4, 5]. Some studies have shown that CRISPR/Cas9 non-specifically cleave DNA sites with several mismatches, generating off-target mutations with considerable frequency [4–9]. The optimal PAM (the yellow part in Fig. 1) recognized by SpCas9 is 5′-NGG-3′. However, SpCas9 also binds 5′-NAG-3′ or 5′-NGA-3′ with low frequency [6, 10]. Accordingly, it is essential to identify potential off-target sites and improve sgRNA specificity.

Finding target sites can generally be accomplished quite easily by scanning the whole genome for the PAM sequence, such as 5′-NGG-3′ for the CRISPR/Cas9 system. Then we can obtain a set *K* of candidates to be sgRNA. However, we must remove the sgRNA with high off-target potential from *K*.

Third-party alignment tools, such as BWA [11] and Bowtie [12], are often used to search off-target sites [13–15]. However, alignment tools are not originally designed for off-target detection. As mentioned in CRISPR-DO [15], for each candidate sgRNA *k-mer* in *K*, alignment tools have to scan the entire genome once to identify its off-target sites, rather than searching in *K*, which results in a large amount of redundant computations. On the other hand, although alignment tools support pattern matching within several mismatches, the mismatch position in the query cannot be set. As a result, these alignment tools cannot consider the PAM sequence when searching mismatched sites. The off-target sites returned by alignment tools contain a number of sequences that are not adjacent to PAM. Accordingly, we have to filter the returned off-target sites and remove those false off-targets [15].

CasOT [16] is a Perl script that searches for potential off-target sites in any given genome, with user-specified PAM. It allows mismatches in the seed and non-seed regions and provides both a single-gRNA searching mode and a paired-gRNA searching mode. CasOT can identify potential off-target sites in an acceptable period of time.

Cas-OFFinder [17] can search for potential off-target sites in a given genome or set of user-defined sequences. It is not limited by the number of mismatches and allows various PAM sequences. The tool is partly written in OpenCL, enabling operations using an accelerator such as GPU, which can significantly speed up the searching process.

Perez et al. constructed a trie to store all candidate sgRNA (*k-mer*) in *K*, and thus to search off-target sites by traversing the trie rather than the entire genome [18]. It is necessary to traverse the trie once for each *k-mer* to detect its off-target sites within several mismatches; consequently, this involves a large amount of redundant computations. In addition, a trie is a space-consuming data structure, and the number of *k-mer*s adjacent to PAM in the genome is usually huge [15]. Detecting off-target sites for large genomes will thus consume hundreds of GB of memory.

Accordingly, in this paper, we present OffScan, a universal and fast CRISPR/Cas off-target site detection tool. OffScan is not limited by a number of mismatches and/or PAM. It adopts FM-index [19] to assist in off-target searching, which efficiently reduces memory footprint and query time and enables the design of highly specific sgRNA.

## Results and discussion

### CRISPR target sites scan

The PAM scan model is used to generate candidate sgRNAs. We tested the PAM scan model of OffScan on four genomes: hg38 (human), mm10 (mouse), danRer7 (zebrafish), and ce10 (*C. elegans*). Since GuideScan [18] does not include a PAM scan model, we used the results of a popular sgRNA design tool, CRISPR-DO [15], for comparison. The total number of *k-mer*s in the millions of target sequences identified by 5′-NGG-3′ is shown in Table 1. OffScan can find a comparable number of candidate target sites compared with CRISPR-DO.

**Table 1:** Total number of candidate target sites in millions

Number (m)	hg38	mm10	danRer7	ce10
CRISPR-DO	303.67	276.57	94.18	7.17
OffScan	304.58	277.36	94.84	7.23

### Off-target detection module

We test the performance of OffScan in hg38 with 1, 3, and 10 mismatches and mm10 with 3 mismatches. The sgRNA length is 20 bp. We arbitrarily chose 1000 SpCas9 targets and run OffScan to detect off-target sites. The CPU version is E7–8890 v3. As shown in Table 2, the number of mismatches and genome size will affect the time taken to detect off-target sites.

**Table 2:** Running of OffScan to search for SpCas9 off-target sites

Data set (size)	Number of mismatches	Time for 1000 targets (s)
hg38 (3.01Gb)	1	552.7 ± 1.5
hg38 (3.01Gb)	3	589.4 ± 2.3
hg38 (3.01Gb)	10	863.5 ± 3.6
mm10 (2.65Gb)	3	462.7 ± 1.8

**Fig. 2:**
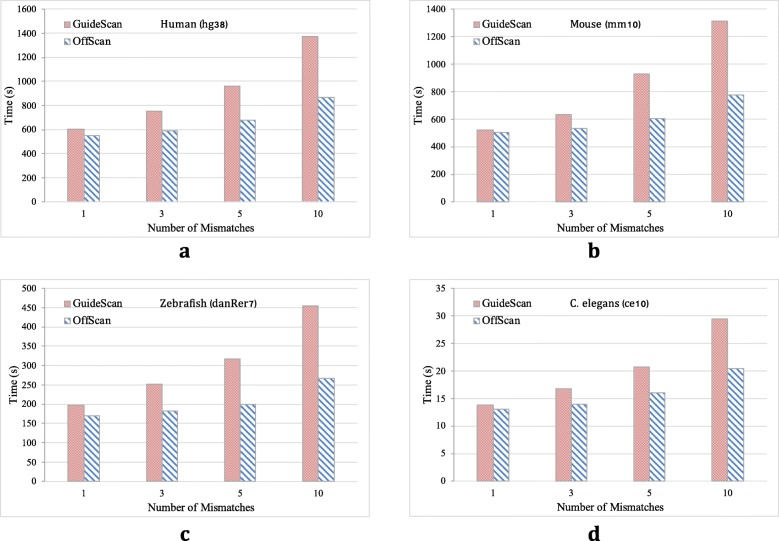
Performance of OffScan and GuideScan

We compared the performance of OffScan with GuideScan under different number of mismatches on four genomes used in the previous test. As shown in Fig. 2, when the number of mismatches is small, the performance is similar. As the number of mismatches increases, the performance gap between the two becomes larger and larger. In the fuzzy matching part, a bounded traversal strategy is adopted. The lower bound of the mismatch number of the query string *Q* and the original string *X* are estimated before the traversal, so that the program can return the traversal of the branch in advance, effectively reducing the search space in suffix tree. The depth of the downward extension increases the efficiency of the comparison. In addition, since OffScan is designed for general sgRNA design, the program behaves similarly on different species.

## Conclusions

OffScan enables off-target site detection in any genome, without limitation of the PAM sequence or number of mismatches. Moreover, OffScan utilizes the FM-index to support fast, memory-efficient, and highly specific sgRNA design. The sgRNA design and off-target site detection can be made more convenient using OffScan. In future, we will incorporate more features into OffScan and provide useful tools for the research community.

## Methods

### CRISPR target sites scan

To facilitate sgRNA design, we implemented a PAM scan module in OffScan. The Cas proteins require a PAM sequence to bind to, so the PAM scan is implemented to scan the entire genome in order to find the sequences with this PAM (e.g. 5′-NGG-3′ for the Cas9 enzyme). The module supports the use of a custom PAM sequence, which can be used for general-purpose sgRNA design. In the majority of situations, we simply need to scan the entire genome once to find the candidate sgRNA with PAM; accordingly, we did not use the FM-index in this module.

### Off-target sites detection

The off-target site detection problem can be divided into two problems: namely, off-target sites with and without mismatches.

We adopt the backward search algorithm [20] to find sites without mismatch, which is a pattern matching problem. Let *∑* be an alphabet. A sentinel symbol $ is not present in it and is lexicographically smaller than all the symbols in ∑. A string *X* = *a*_*0*_*a*_*1*_ *...a*_*n − 1*_ is terminated with symbol $ (i.e. *a*_*n − 1*_ = $) and this symbol only appears at the end. The length of string *X* is |*X*| = *n*. *X*[*i*] = *a*_*i*_ is the *i-*th symbol of *X* and *X*[*i,j*] is the substring *a*_*i*_…*a*_*j*_.

**Fig. 3:**
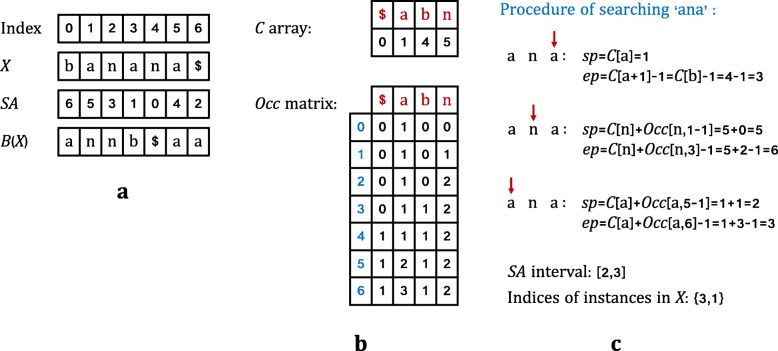
FM-index. **a** Suffix array and BWT of string *X*. **b** *C* array and *Occ* matrix in FM-index. **c** The procedure of backward search algorithm of searching ‘ana’ in ‘banana’

The suffix array data structure is a succinct representation of the lexographic ordering of all the suffixes of a string [21]. The suffix array of string *X*, denoted as *SA*(*X*), is actually the permutation of the indices {1, 2, …, n-1} of *X* that

1 $$ SA\left[i\right]=j $$

iif *X*[*j,n*-*1*] is the *i*-th lexographically smallest suffix of *X*. For example, if *X* = banana$, the *SA*(*X*) = [6,5,3,1,0,4,2], as shown in Fig. 3a. Since the suffixes in *SA* are sorted in lexographic order, the start positions of all the instances of a pattern *Q* in *X* should be an interval of *SA*, that is called a suffix array interval, denoted as a pair of integers [*sp*,*ep*]. Thus, the pattern matching of *Q* in *X* is equivalent to find the suffix array interval of *Q* in *X*.

Ferragina and Manzini developed a data structure called FM-index [19], which can determine the suffix array interval [*sp*,*ep*] of pattern *Q* in *O*(|*Q*|) time and requires much less memory than a suffix array. FM-index is a careful combination of a compression algorithm Burrows-Wheeler transform (BWT) [22] and suffix array. The BWT of *X*, denoted as *B*(*X*), is a permutation of the string that

2 $$ B\left[i\right]=\left\{\begin{array}{c}X\left[ SA\left[i\right]-1\right], SA\left[i\right]>0\\ {}\$, SA\left[i\right]=0\end{array}\right. $$

That is to say, *B*[*i*] is the symbol preceding the first symbol of the suffix starting at *SA*[*i*]. For example, if *X* = banana$, the *B*(*X*) = annb$aa, as presented in Fig. 3a.

To enable pattern matching with FM-index, Ferragina and Manzini added two more data structures: *C* array and *Occ* matrix. As illustrated in Fig. 3b, for a symbol *a*, *C*[*a*] is the number of occurrences of symbols that are lexographically smaller than *a*. *Occ*[*a,i*] records the number of occurrences of the character *a* in *B*[0,*i*]. For a query *Q* whose suffix array interval in *X* is [*sp*,*ep*], the interval of string *aQ* can be calculated with *C* and *Occ* arrays as follows:

3 $$ {\displaystyle \begin{array}{c} sp\left[ aQ\right]=C\left[a\right]+ Occ\left[a, sp\left[Q\right]-1\right]\\ {} ep\left[ aQ\right]=C\left[a\right]+ Occ\left[a, ep\left[Q\right]\right]-1\end{array}} $$

From the above eq. (3), we notice that the pattern matching should start from the last character of *Q*, that is backward search algorithm. Algorithm 1 presents the details of searching procedure. Firstly, the suffix array interval of the last symbol of *Q* is calculated from *C* array. Then the interval is calculated iteratively based on Eq. (3), as shown in Fig. 3c. For the returned values of backward search algorithm, if *sp* < *ep*, *Q* has more than one instances in *X*; if *sp* = *ep*, *Q* has just one instance in *X*; if *sp* > *ep*, *Q* is not included in *X*.

**Figure:**
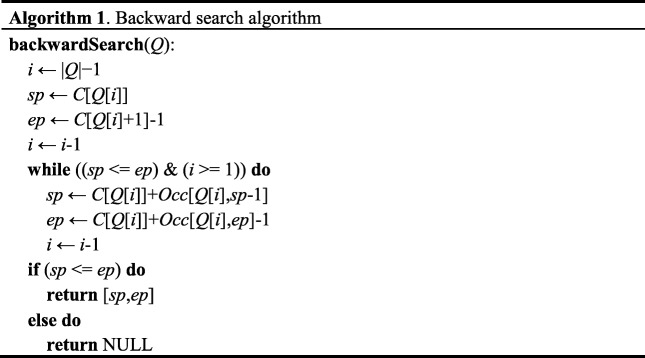

The backward search algorithm updates the suffix array interval at most |*Q*| times, so the time complexity is *O*(|*Q*|). The time complexity of backward search is linear of query string length |*Q*|, equal to the exact matching in a trie; however, the FM-index is more space-efficient than a trie. Besides, the backward search algorithm actually realizes a top-down traversal of a trie.

**Figure:**
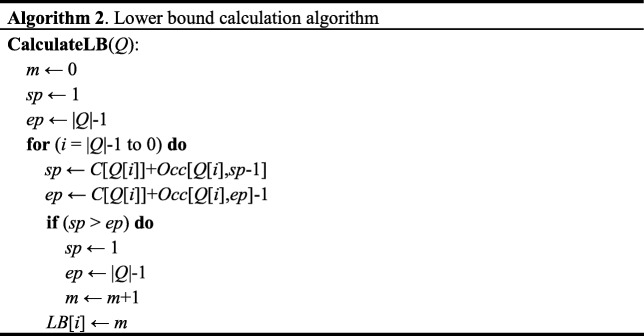

Since sgRNA allows several mismatches when binding to DNA, an off-target detection algorithm supporting only exact match is not sufficient. OffScan also implements search with mismatches. A naïve method is to traverse the trie to search sgRNA with mismatches, but this process is rather time-consuming. To avoid unnecessary comparisons, we introduce a bounded-search for mismatches in OffScan. For a query of *Q* in *X*, we define *LB*[*i*] as the lower bound of the number of differences between *Q*[*i*,|*Q*|-1] and *X*. If the current allowing number of mismatches is smaller than *LB*[*i*], the search process will stop and backtrack to other branches. Algorithm 2 presents the procedure of calculating *LB*[*i*].

### FM-index construction

The off-target site detection module used in OffScan supports any number of mismatches, including no mismatches for exact matching. In terms of the general procedure, we have to detect off-target sites for each *k-mer*. To avoid the redundant computations involved in scanning of the entire genome, we detect off-targets only within the candidate sgRNA set *K.*

To improve the query efficiency and reduce space requirements, we adopt the FM-index to aid in off-target site detection. While this data structure is originally designed for single-string queries, we want to extend it to multiple sequence queries. To solve this problem, we add a “$” character to the end of each *k-mer* and concatenate all *k-mer*s as a long string. We then construct an FM-index for the concatenation.

## Data Availability

Please contact author for data requests.

## Abbreviations

BWT: Burrows-Wheeler transform
Cas: CRISPR-associated
CRISPR: Clustered regularly interspaced short palindromic repeats
DSB: Double-strand break
PAM: Protospacer-adjacent motif
sgRNA: Single guide RNA
